# COVID-19 Isolation Control Proposal *via* UAV and UGV for Crowded Indoor Environments: Assistive Robots in the Shopping Malls

**DOI:** 10.3389/fpubh.2022.855994

**Published:** 2022-05-31

**Authors:** Muhammet Fatih Aslan, Khairunnisa Hasikin, Abdullah Yusefi, Akif Durdu, Kadir Sabanci, Muhammad Mokhzaini Azizan

**Affiliations:** ^1^Electrical and Electronics Engineering, Karamanoglu Mehmetbey University, Karaman, Turkey; ^2^Department of Biomedical Engineering, Faculty of Engineering, Universiti Malaya, Kuala Lumpur, Malaysia; ^3^Center of Image and Signal Processing (CISIP), Faculty of Engineering, Universiti Malaya, Kuala Lumpur, Malaysia; ^4^Computer Engineering, Konya Technical University, Konya, Turkey; ^5^Electrical and Electronics Engineering, Konya Technical University, Konya, Turkey; ^6^Department of Electrical and Electronic Engineering, Faculty of Engineering and Built Environment, Universiti Sains Islam Malaysia, Bandar Baru Nilai, Malaysia

**Keywords:** COVID-19, HOG, SegNet, semantic segmentation, Support Vector Machine, UAV

## Abstract

Artificial intelligence researchers conducted different studies to reduce the spread of COVID-19. Unlike other studies, this paper isn't for early infection diagnosis, but for preventing the transmission of COVID-19 in social environments. Among the studies on this is regarding social distancing, as this method is proven to prevent COVID-19 to be transmitted from one to another. In the study, Robot Operating System (ROS) simulates a shopping mall using Gazebo, and customers are monitored by Turtlebot and Unmanned Aerial Vehicle (UAV, DJI Tello). Through frames analysis captured by Turtlebot, a particular person is identified and followed at the shopping mall. Turtlebot is a wheeled robot that follows people without contact and is used as a shopping cart. Therefore, a customer doesn't touch the shopping cart that someone else comes into contact with, and also makes his/her shopping easier. The UAV detects people from above and determines the distance between people. In this way, a warning system can be created by detecting places where social distance is neglected. Histogram of Oriented-Gradients (HOG)-Support Vector Machine (SVM) is applied by Turtlebot to detect humans, and Kalman-Filter is used for human tracking. SegNet is performed for semantically detecting people and measuring distance via UAV. This paper proposes a new robotic study to prevent the infection and proved that this system is feasible.

## Introduction

A novel coronavirus family called COVID-19 spread around the world, starting in China as a respiratory infection in late 2019. Because of the rapid spread of this disease and its accompanying symptoms, the World Health Organization (WHO) announced COVID-19 as a pandemic. COVID-19 caused severe problems to all as business and social activities were shut, therefore resulted in economic downturn and people had to live in house. Currently, worldwide, the total number of cases and death exceeded 95 million and 2 million, respectively. In the current period, many researchers have carried out studies such as early detection of the disease, reducing its effects, and its effects on human life. Most past studies aimed to successfully identify people infected with COVID-19 as early as possible so they can be quarantined and treated quickly (1). Due to the insufficient and unreliability of the Reverse Transcription Polymerase Chain Reaction (RT-PCR) test, the detection of COVID-19 through medical images has come to the fore. In particular, disease detection based on medical images has recently gained importance after chest Computed Tomography (CT), as scanning procedure has performed better against RT-PCR in negative or weakly positive RT-PCR cases (2, 3). In addition to CT images, successful results have been obtained for COVID-19 testing with scanning images such as chest X-Ray and Magnetic Resonance Imaging (MRI). These images label the medical image as positive/negative by using Artificial Intelligence (AI)-based Deep Learning (DL) or Machine Learning (ML) algorithms (4–9).

These early diagnosis studies conducted so far aimed to assist medical experts. However, the contributions that can be made against the COVID-19 crisis are not limited to these biomedical studies. In order to prevent the spread or transmission of this infection, we need to control our social life. However, life of all people is impossible to control, and therefore the transmission of diseases occurred hence the spread of COVID-19 to the mass. To overcome this, many countries started initiatives to limit social activities, to the extent the suspension of businesses and places of commons, which resulted economic crisis. In addition, distance education and flexible working policy have been adopted by many countries. Although vaccination applications have begun for this epidemic that has been going on for about 2 years, it is highly likely that COVID-19 will still be in our lives for a long time due to the mutation of the virus and the continuing uncertainty about the vaccine. For this reason, we need to spend our lives in accordance with the rules of cleanliness, hygiene and distance as long as the coronavirus exists. Service robots or social robots can help people to provide these rules in our social life (10).

### Artificial Intelligence and Robotic Solution for COVID-19

Today, with the rapid development in robotic, AI and automation fields, it is estimated that service robots will exist more in our lives and we will share the same environment with robots. Because mobile robots can replace people in surveillance (11), exploration (12), search and rescue (13), entertainment (14), tour guide (15), airport (16), medical (17), etc. and can perform these tasks flawlessly (18, 19). Applications that can perform human-like tasks can be developed with mobile robots; this depends on thinking like a human and interpreting what they see like a human. Such applications have now become possible with advancements in computer vision and AI applications. AI-based ML and DL techniques have proven their potential, effectiveness and versatility, proving their success in several areas, particularly computer vision. Data-driven DL methods, especially using Convolutional Neural Networks (CNNs), have achieved a high degree of success in pattern recognition and object detection in the last decades. With CNN, features are extracted from input data (usually image data), and training and classification are performed with these features. In this sense, it differs from ML methods as it provides both feature extraction and classification. In addition, its generalization ability is superior to traditional ML methods (20). A network architecture is first designed for feature extraction and classification/regression applications with CNN. Popular CNN models such as GoogLeNet (21), VGG-Net (22), ResNet (23), Inceptionv3 (24), etc., which have proven their success in the past, are still frequently used today. These models take different types of images as input and estimate or classify them according to the extracted features.

For the development of mobile robots that share the same environment with humans, many tasks must be performed simultaneously in real-time. In general, the most frequently used robots today can be specified as wheeled robots and Unmanned Aerial Vehicle (UAV). Especially recently, UAVs have enabled the development of technologies that provide significant convenience in human life and, therefore, attracted researchers' attention (25). In real-time mobile robotic applications, object location and object tracking are important as well as object detection. In order to achieve this, the object detection and location are predicted with the features extracted from the frame, then a tracking algorithm is performed using the current position of the object. Therefore, the object must be successfully distinguished from the environment for a strong tracking. Traditionally, contour-based methods have been a popular choice in the past for high-quality image segmentation. However, in recent years, especially data-driven DL-based segmentation methods have become popular due to their impressive performance (26). In advanced DL-based applications, the object can be detected semantically. These studies, called semantic segmentation, are especially important for autonomous vehicles (27), video surveillance (28) and augmented reality (29) applications. Semantic segmentation, which solves image segmentation as pixel classification, is preferred because they are completely automatic and perform segmentation at the pixel level (30). Unlike other methods such as image classification and object detection, semantic segmentation can also calculate the boundaries and position of the object in addition to the category, size and quantity of the target object. On the advantages it provides, many DL-based semantic segmentation methods such as FCN (31), U-Net (32), SegNet (33), DeepLabv3 (34), DeepLabv3+ (35), etc. have been proposed (36). These generally adopt the encoder-decoder structure.

In most of the previous studies (37–40) where AI and computer vision are applied to combat COVID-19, the aim is to detect infection from scan images. Developing these studies with AI techniques based on image data is relatively easier than robotic applications. Therefore, when previous studies are examined, it is seen that the number of robotic solutions developed for the COVID-19 crisis is low. Kimmig et al. (41) emphasize the importance of robot-assisted surgery during the COVID-19 pandemic. Feil-Seifer et al. (42) discussed the impact of COVID-19 on Human Robot Interaction (HRI) studies. They stated that HRIs will be needed more after COVID-19. Li et al. (43) noted that oropharyngeal swab (OP swab) sampling is widely used to diagnose COVID-19. They recommended the Robotic Sampling (RS) system to prevent close contact between healthcare professionals and patients during OP swab application. Similarly, Wang et al. (44) emphasized that nasopharyngeal (NP) swab sample collection poses a risk of infection for healthcare personnel who are in close contact with the suspected patient. To address this risk, the authors developed a remotely controlled low-cost robot to assist with NP sampling. In another study, Wei et al. (45) proposed a COVID-19 detection system using a robot. The aim was to provide the detection of COVID-19 with speech, cough and temperature measurement data by providing dialogue between humans and robots. In a different study, the humanoid robot Pepper was designed to help doctors communicate remotely with their patients and avoid contact without being in the same room (46).

The above studies show the importance of robotic solutions to reduce the impact of the COVID-19 pandemic. However, studies in this area are new and need to be developed. In addition, the above studies conducted do not aim to reduce the effect of the coronavirus that people are exposed to in their social life. To reduce the rate of spread of COVID-19, experts constantly warn people about social distancing and contact. However, since social distance and contact rules cannot be controlled in crowded environments, the virus spreads rapidly. It is not possible to constantly monitor the rules by a person in crowded environments and to intervene in case of violation. For this, robotic systems and modern AI techniques can help us.

There are also studies carried out to prevent the spread of infection in social life. In one of these studies, Shao et al. (47) stated that UAVs will be highly preferred for social distance monitoring in the future. For this reason, they developed a human head detection system with a UAV using deep PeleeNet architecture, thereby detecting pedestrians in real-time. Finally, they calculated the social distance between pedestrians from the UAV images. In another study, Punn et al. (48) proposed a real-time application based on DL to automate social distance tracking. They used YOLO v3 to detect and mark people and the Deepsort method to track detected people. Yang, et al. (49) conducted a deep learning-based study that can detect social distance violations. They used Faster R-CNN and YOLO v4 for human detection. This study was a real-time application that could send audio-visual cues in case of violation. Finally, Rezaei and Azarmi (50) developed a deep architecture model named DeepSOCIAL based on YOLO v4 for human detection, tracking and distance estimation for automatic human detection using CCTV security camera images in indoor and outdoor crowded environments.

All of these studies aimed to automatically detect the social distancing that people tend to violate. Therefore, UAVs are an important tool for such applications, especially with their flexible mobility. These studies show that tools such as UAV, AI, computer vision can play an important role in reducing the spread of COVID-19. Generally speaking, mobile robots can calculate distances between people and thereby control the spread of the infection (42). In this sense, shopping malls, which are crowded and a place where social distance cannot be maintained, are important in terms of the risk of transmission of the coronavirus. Although our study is similar to the above studies measuring social distance, it also contains important differences. Our study proposes a multi-robot system consisting of a wheeled robot (or service robot) and a UAV to reduce the spread of COVID-19 in a crowded shopping mall environment. The wheeled robot follows the human within a safe distance range and is used to reduce human contact. In this way, customers do not touch the shopping cart that someone else has touched. The UAV, on the other hand, calculates the social distance between people, similar to previous studies. This study is the first to prevent contact-based virus transmission with the shopping cart scenario. Moreover, since it includes a multi-robot application that handles contact and social distance at the same time, it will make significant contributions to future studies.

### Purpose and Contributions of This Paper

This study suggests a service robot and UAV application in order to reduce the transmission of infection between individuals during the COVID-19 period. The aim is to reduce contact and maintain distance between people in a wide area where people are collectively and shopping. For this, a shopping center environment with a ground robot and a UAV is simulated in the Robot Operating System (ROS) Gazebo environment. The ground robot is thought of as a shopping cart that follows the human through its front camera. In one of our previous studies^1^ (51) we designed a shopping robot that follows people. The same purpose applies to this study. This robot, which carries the materials bought by the customers, also prevents human contact due to the fact that it is a shopping cart. In this way, the risk of COVID-19 transmission is reduced due to reduced human contact. To achieve this implementation, the robot must be able to successfully identify the customer and then follow her/him. This study applies the Histogram of Oriented Gradients (HOG)-Support Vector Machine (SVM) feature extraction and classification technique for human detection. In our previous study aimed at human tracking (20), different methods such as Kalman Filter (KF), Particle Filter (PF), Kalman – Particle Filter (KPF) were used and detailed information for human tracking was shared. Similarly, KF-based human tracking is performed in this study.

Regardless of the ground robot that follows the human, for social distance detection, the semantic detection of humans is performed with images taken from a sub-camera of the UAV. SegNet architecture is used for the semantic segmentation of the people in the environment. SegNet architecture is first trained with Semantic Drone Dataset (52), then, the frames that we produce in Gazebo are given to the trained architecture. As a result, people in the environment can be distinguished based on the UAV images. Afterward, the distance between persons can be calculated. Since the distance between people cannot be calculated reliably using the camera of the ground robot, the images taken from the UAV calculate this distance more successfully and easily. In this way, a warning system is created by identifying areas where the social distance between people is neglected. As a result, our study aims to both eliminate contact and maintain social distance, which are the two most important elements to reduce the transmission rate of COVID-19.

Our study differs from previous studies in terms of preventing social contact with the shopping service robot, measuring social distance through semantic segmentation from UAV images, and simulating it in a shopping mall environment. The contributions that make this study different from previous studies can be summarized as follows:

1) The study suggests a robotic study to be used in closed and crowded shopping malls to reduce the transmission rate of COVID-19.
2) A simulation is performed in the ROS Gazebo environment for the proposed study.
3) Both ground robot and UAV are used.
4) Thanks to the ground robot, people do not touch the shopping cart, the ground robot follows people in a safe range.
5) With UAV images, people are detected semantically and social distance between people is measured.

The rest of the paper is organized as follows. Section Datasets explains the public dataset used for semantic segmentation of UAV images and Gazebo simulation data we have produced. The proposed methodology, application and results are mentioned in section Experimental Studies and Results. Section Conclusion concludes and evaluates this study. Finally, section Discussion and Future Works explains its shortcomings and future plans.

## Datasets

In this section, the datasets used in this paper are introduced.

### Semantic Drone Dataset

**Figure 1:**
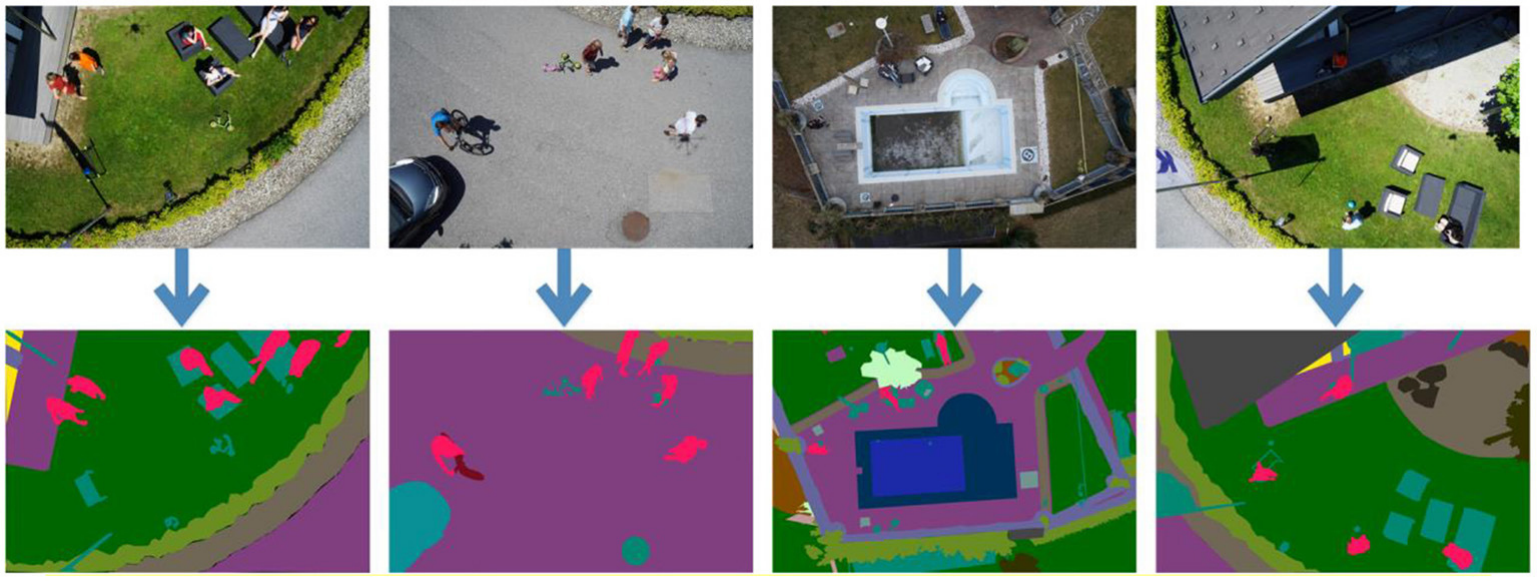
Sample Semantic Drone Dataset images and corresponding semantic masks (Different classes in each image are marked with different colors) (52).

Semantic Drone Dataset (52) contains video frames recorded via a UAV and semantic masks of certain objects in these frames. Images were recorded from a bird's eye view at a range of 5–30 meters. A high-resolution camera (24MP, 6,000 × 4,000) was used to capture images. There are 600 images in total and 200 of them are reserved for training. Semantic images consist of a total of 20 classes such as trees, bicycles, walls, fences, doors, pools, rocks, dogs, cars, etc. Figure 1 shows some UAV images of the Semantic Drone Dataset and the corresponding semantic mask images.

### Gazebo Shopping Malls Dataset

A shopping malls simulation environment spanning 20 × 25m and containing different simulated objects and humans prepared in Gazebo (53) has been used to collect datasets. The Gazebo is an open-source library designed to simulate the real world. All implementations have been performed on the ROS^2^ framework using the python programming language. We collect datasets from the same environment using two different simulated robot models, a ground robot and an aerial robot.

The simulated ground robot used was the Turtlebot 2 model with a Kinect depth camera. It was used to collect a ground view of the shopping malls environment and the people inside it. This dataset was later used for human detection and tracking tasks. The data set collected by this ground robot included time-stamped image depth data, RGB image data, IMU data, and odometry data. RGB and depth images were captured at a resolution of 640 x 480 pixels, 30 Hz, and IMU and ground truth odometry data at a frequency of 300 Hz.

**Figure 2:**
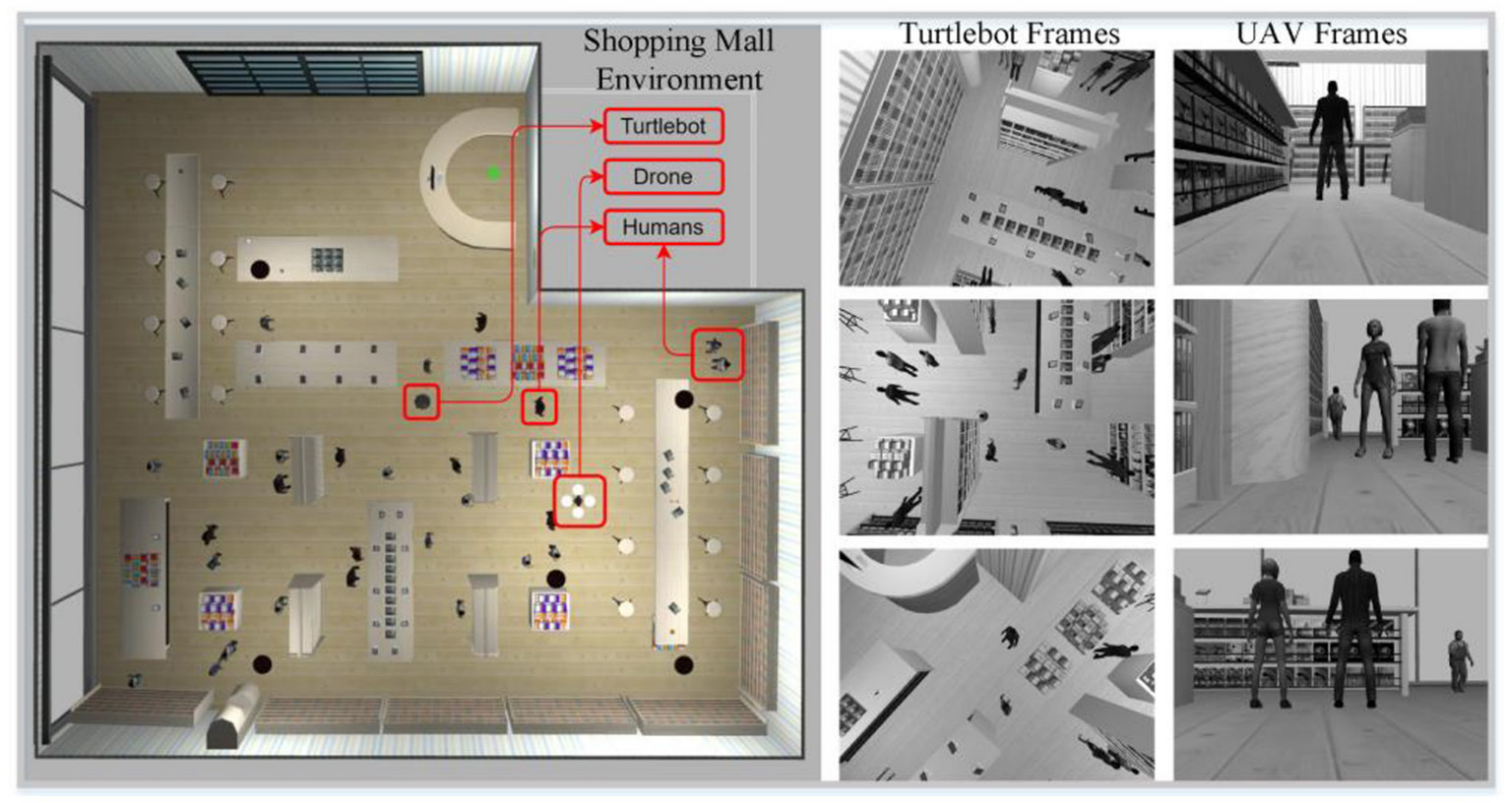
Top view of the designed shopping mall simulation environment including Turtlebot and UAV, and some frames acquired by Turtlebot and UAV (Turtlebot and UAV move independently of each other in the shopping center and perform their tasks through the frames they obtain).

The aerial robot used was a simulated drone quadrotor equipped with a downward camera installed on the bottom of the drone. This drone was used to collect a top view dataset of the environment and the people in it. This dataset was later used to detect humans in the shopping malls and to calculate the distance between them. The dataset collected by this aerial robot included gray-scale images, biased IMU data, unbiased IMU data, and ground truth odometry data. Gray-scale images with a resolution of 640 x 480 and a frame rate of 30 FPS were collected. Same as the ground vehicle, IMU and ground truth data on drones were collected at a frequency of 300 Hz. Figure 2 shows the shopping mall simulation environment, Turtlebot 2 and UAV in the environment, and some frames captured by both robots.

## Experimental Studies and Results

In this section, information is given about the applications performed with the ground robot and UAV and the results obtained.

### Human Tracking *via* Wheeled Robot in Shopping Malls

**Figure 3:**
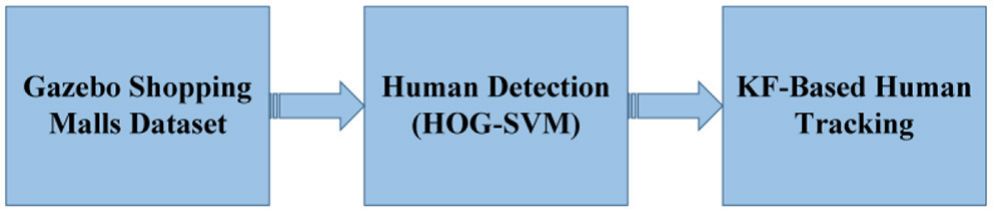
Application steps of human tracking with the ground robot (Using the frames obtained by Turtlebot moving in the Gazebo Shopping Malls environment, feature extraction and human detection are applied with the help of HOG-SVM. As a result, humans in the environment are detected and marked. Finally, KF-based human tracking is performed, which provides the next position estimation of the human).

This section basically consists of two applications (see Figure 3): (i) HOG feature extraction method and SVM classification method for detecting people in frames produced in Gazebo, (ii) KF-based tracking method to follow the detected person in the next frames.

**Figure 4:**
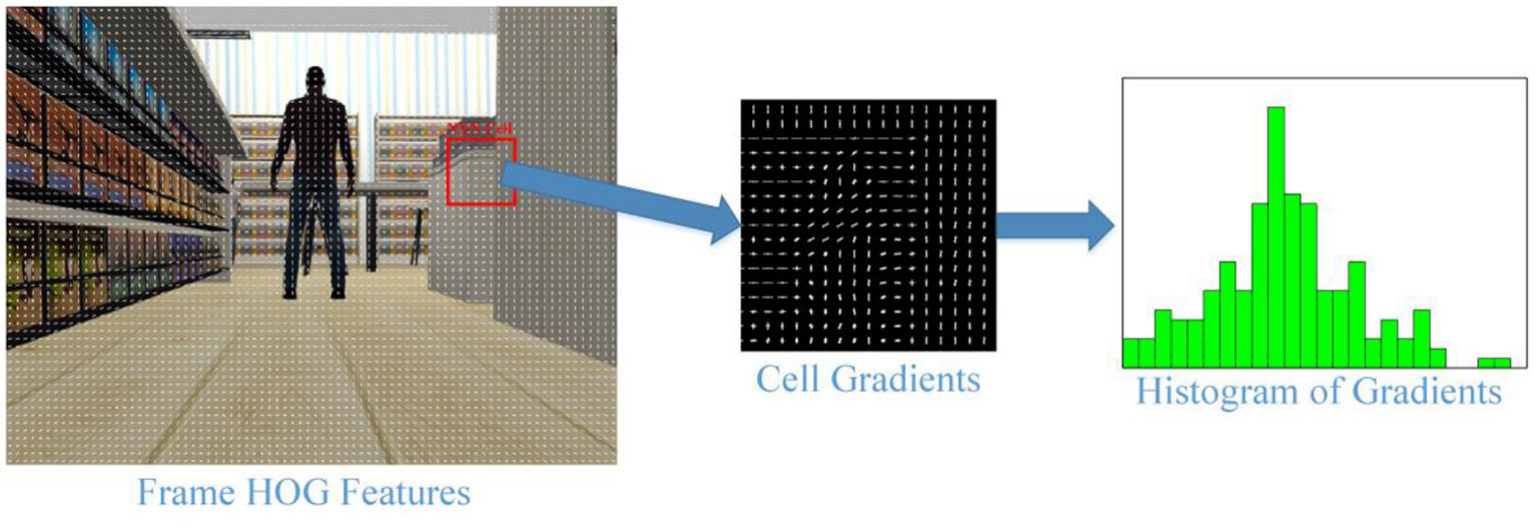
Gradients for a cell of a sample Turtlebot frame and generating HOG features from these gradients.

HOG (54) is a feature extraction method that best represents the geometric structure of the human in an image. Robust features extracted from a frame for human detection are then given to the prediction algorithm. SVM is generally good for predicting and is therefore often preferred (HOG-SVM) (54, 55). HOG descriptors are frequently applied in computer vision studies and perform object detection depending on the shape. In the HOG algorithm, first the image is divided into cells of *N* × *N* pixels, then the gradients of each cell are calculated. Histograms are created to take advantage of the distribution of these gradients. All histograms are combined, transforming them into a 9-channel row matrix. This feature vector represents the image. In this context, HOG stands out as a powerful shape recognition algorithm. Figure 4 shows the gradients and histograms of an *N*× *N* cell as a result of applying HOG to an image of the Gazebo Shopping Malls Dataset. In our study, it is determined as *N* = 8.

**Figure 5:**
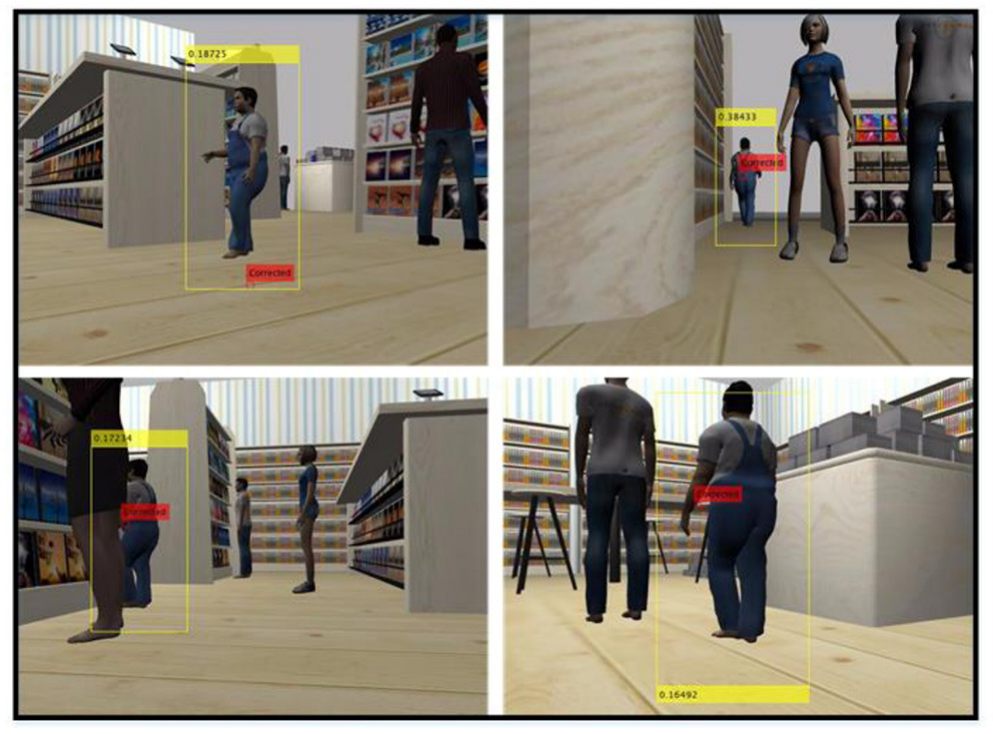
Tracking of a customer in the mall with KF after human detection is completed with HOG-SVM.

The HOG algorithm shown in Figure 4 is applied to all frames taken from the front camera of the ground robot. Feature vectors extracted from each frame are given to the SVM algorithm and human detection is performed. The borders of the detected person are then marked with a bounding box (see Figure 5). The values in the bounding box represent the degree of confidence of the detection.

In order for the robot to follow the human, the next step after human detection is the tracking algorithm. In this way, people whose borders are determined by the bounding box can be followed. This study uses a statistical-based Bayesian method to track humans. The most important Bayesian-based state estimation methods are undoubtedly KF and Particle Filter (PF). Xu and Gao (56) applied the HOG-SVM method for human detection. However, for human tracking, the Bayesian-based PF was utilized. In a different study conducted by Li et al. (57) and Ma et al. (58). HOG was used together with KF. Also, in our previous study (20), the most successful human tracking was achieved with KF.

In order to follow an object in an image with KF, state information resulting from object movements and measurement information obtained by observing the object are combined. In other words, a prediction is made first (predict), and then the prediction is verified by measurement (update). This process continues iteratively throughout the video with the predict-update steps. Since Bayesian state estimation methods make estimates using measurement and state uncertainties, they are suitable for real world studies and are very fast. For KF-based tracking, the values for human detection (position, speed, etc.) obtained by HOG are measurement information, while physical calculations based on human movement (see Equations 1 and 2) are state information. The variables for Equations (1) and (2) are as follows: *t*; frame time, *x*; object position, *v*; object velocity and *a*; acceleration. As long as the KF-based tracking continues, these calculations are made at every iteration, this step is also called the prediction step. The next step where measurements and verification are carried out is also known as the measurement step.


(1) $$\begin{array}{rcl} x_{t} & = & x_{t-1}+v_{t-1}t+\frac{1}{2}at^{2} \end{array}$$

(2) $$\begin{array}{rcl} v_{t} & = & v_{t-1}+at \end{array}$$

**Figure 6:**
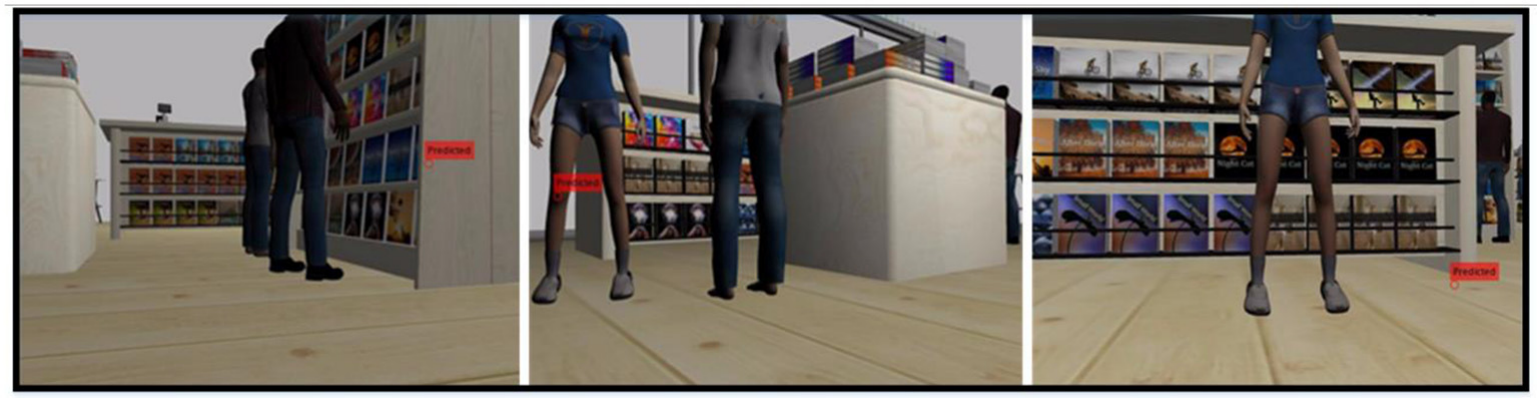
The prediction made by KF in frames where people cannot be detected by HOG-SVM (In cases where human detection cannot be made, predictions made by KF cannot be corrected with the measurement value. This causes erroneous estimations).

As a result of applying KF-based tracking to our dataset, the tracking of a detected customer is shown in some frames in Figure 5. As a result of detection and tracking, the human can be followed by the ground robot. In the frames shown in Figure 5, the human position is labeled as “Corrected” since the prediction-measurement steps are performed in the tracking process with KF after human detection. That is, in the frames where the human position is calculated with HOG-SVM, the calculated physical position estimation is corrected. However, during the tracking algorithm, people may not be detected due to occlusion, blur, noise, etc. In this case, KF can predict the human position, but cannot correct it. In cases where humans cannot be detected, frames for which predictions were made based on previous human positions are given in Figure 6. Predictions that cannot be corrected by measurement (HOG-SVM) drift over time, producing meaningless values. Therefore, the estimated human position deviates from its true value unless corrected by measurement using KF. In this case, the ground robot moves toward the predicted point. If the robot detects the human with the HOG-SVM in the next frames, the KF will quickly approach the true position value again. In general, when the detection and tracking algorithm results are examined, it is seen that the ground robot can follow people and thus human contact with the shopping carts can be prevented.

### Human Semantic Segmentation and Social Distance Measurement *via* UAV in Shopping Malls

**Figure 7:**
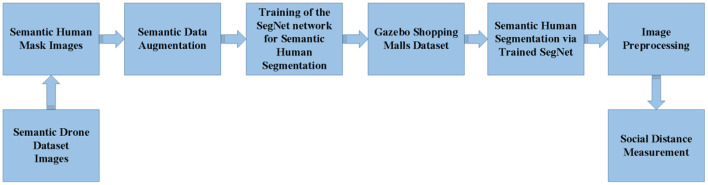
Application steps of human semantic segmentation using UAV (First, the SegNet architecture is trained with the public dataset. Then the trained SegNet architecture is tested on our dataset. After the human detection, the social distance between people is determined by image processing).

This section describes human detection using UAV images taken from a shopping center environment. Application steps are shown in Figure 7. Human detection is required for social distance measurement. However, human detection using UAV images is not as easy as in the ground robot. Because the human geometry varies a lot in UAV images. Sometimes they can be round like a point and sometimes have a shape that cannot be defined. For this reason, this study uses semantic segmentation for a stronger detection. The most advanced way to distinguish an object from an environment is semantic segmentation. In this way, the target object is segmented from the environment (or background) in a human-like manner. Therefore, semantic segmentation has attracted increasing attention in recent years. Semantic segmentation classifies each pixel in the image using deep architectures.

**Figure 8:**
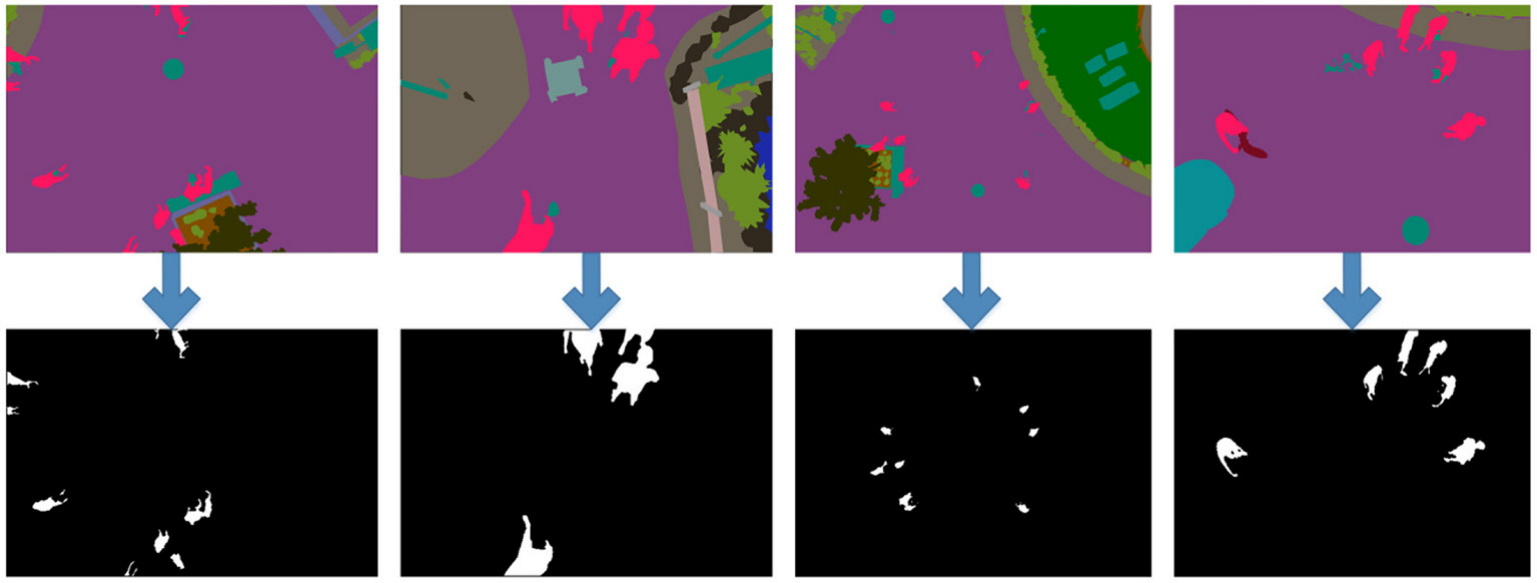
Conversion of 20-classes semantic mask images to 2- classes (The number of classes has been reduced to 2 since our study aimed to only recognize human and non-human pixels).

**Figure 9:**
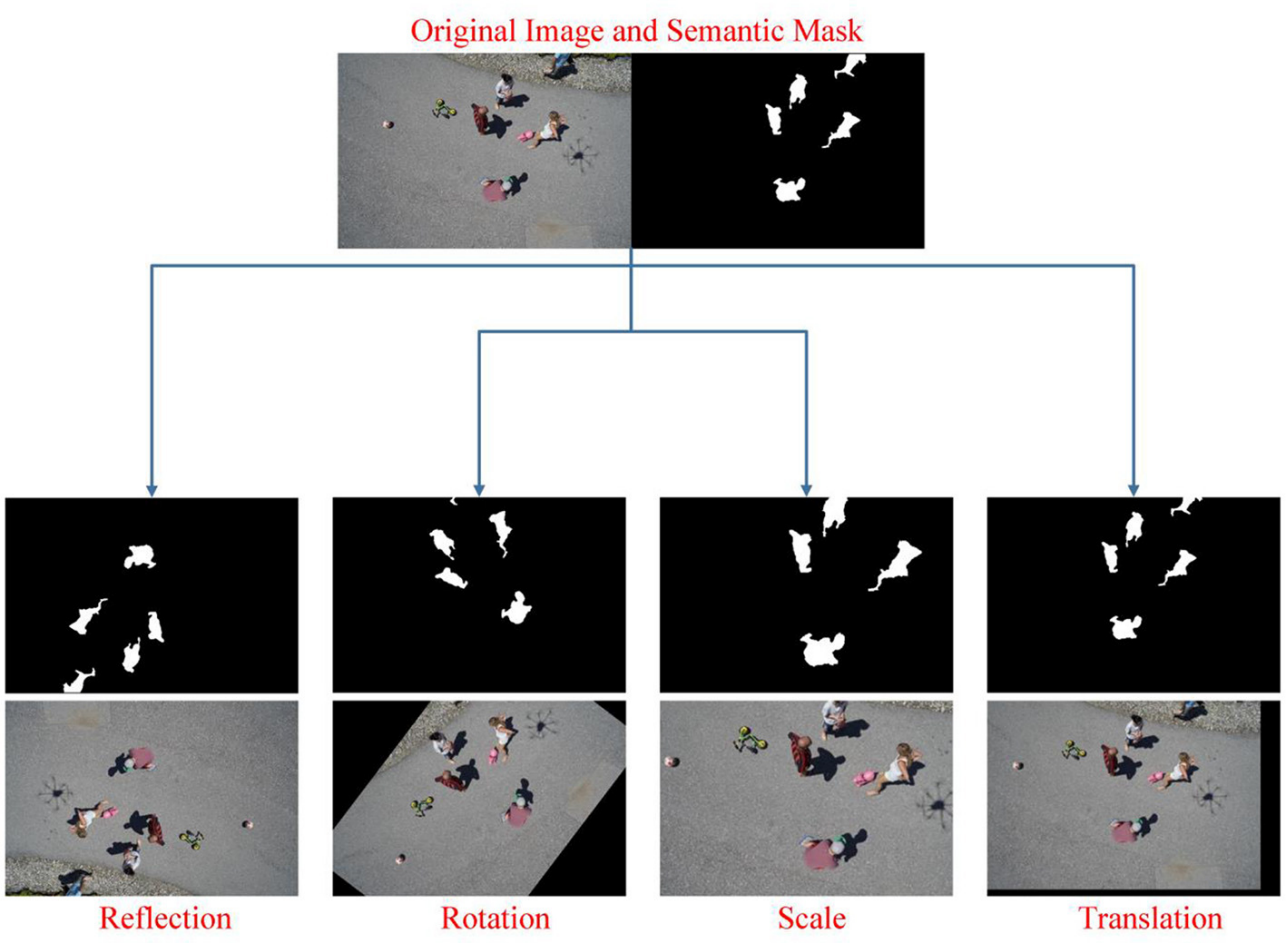
Demonstration of the data augmentation methods used on a sample RGB and mask images (The number of both RGB and mask images is increased for more robust training of the Segnet network).

In traditional CNN networks, stride and pooling operations cause a reduction in output size, so they cannot generate object boundaries for semantic segmentation. To solve this, architectures such as SegNet, U-Net, etc. using the encoder-decoder structure have been proposed. While U-Net combines feature maps in corresponding scales in encoder and decoder architectures, SegNet stores maxpooling indices in the encoder path. Due to the learnable upsampling in its structure, U-Net has many more parameters to learn during the training phase. Therefore, training of U-Net is relatively slow compared to SegNet (59). Moreover, in the study by Manickam et al. (60), in which semantic human detection from UAV images was made, SegNet provided more successful results than many other deep models. Similarly, in another study for brain tissue segmentation by Kumar et al. (59), SegNet provided more successful classification than U-Net. As a result, in this study, SegNet architecture was preferred for semantic human detection from UAV images. However, SegNet, which has a deep architecture, requires large and labeled data. This study uses Semantic Drone Dataset for this. However, the semantic dataset does not contain sufficient samples and has a large number of classes in addition to humans. For this reason, in order to modify the data according to our study, the number of classes is reduced first. In order to perform a more successful segmentation than this public data consisting of 20 classes in total, the number of classes is reduced to 2. Figure 8 shows the 2-class equivalents of 20-class some semantic images. In this way, only human pixels are recognized by the network. However, 600 data in total may not be sufficient for the network to learn. Therefore, data augmentation is applied for 2-class semantic images to provide data diversity and increase success. 4 different data augmentation techniques are used. The lower and upper limit values of these data augmentation methods are shown in Table 1. In addition, new images produced as a result of applying data augmentation techniques to an original and two-class image are shown in Figure 9.

**Table 1:** Data augmentation techniques and lower-upper limit values of each technique (In the data augmentation step, random values are determined in the specified range for each data augmentation technique).

**Parameter name**	**Lower limit**	**Upper limit**
Reflection	-	-
Rotation	−60°	60°
Scale	1.1	1.5
Translation (pixel)	−50	+50

**Figure 10:**
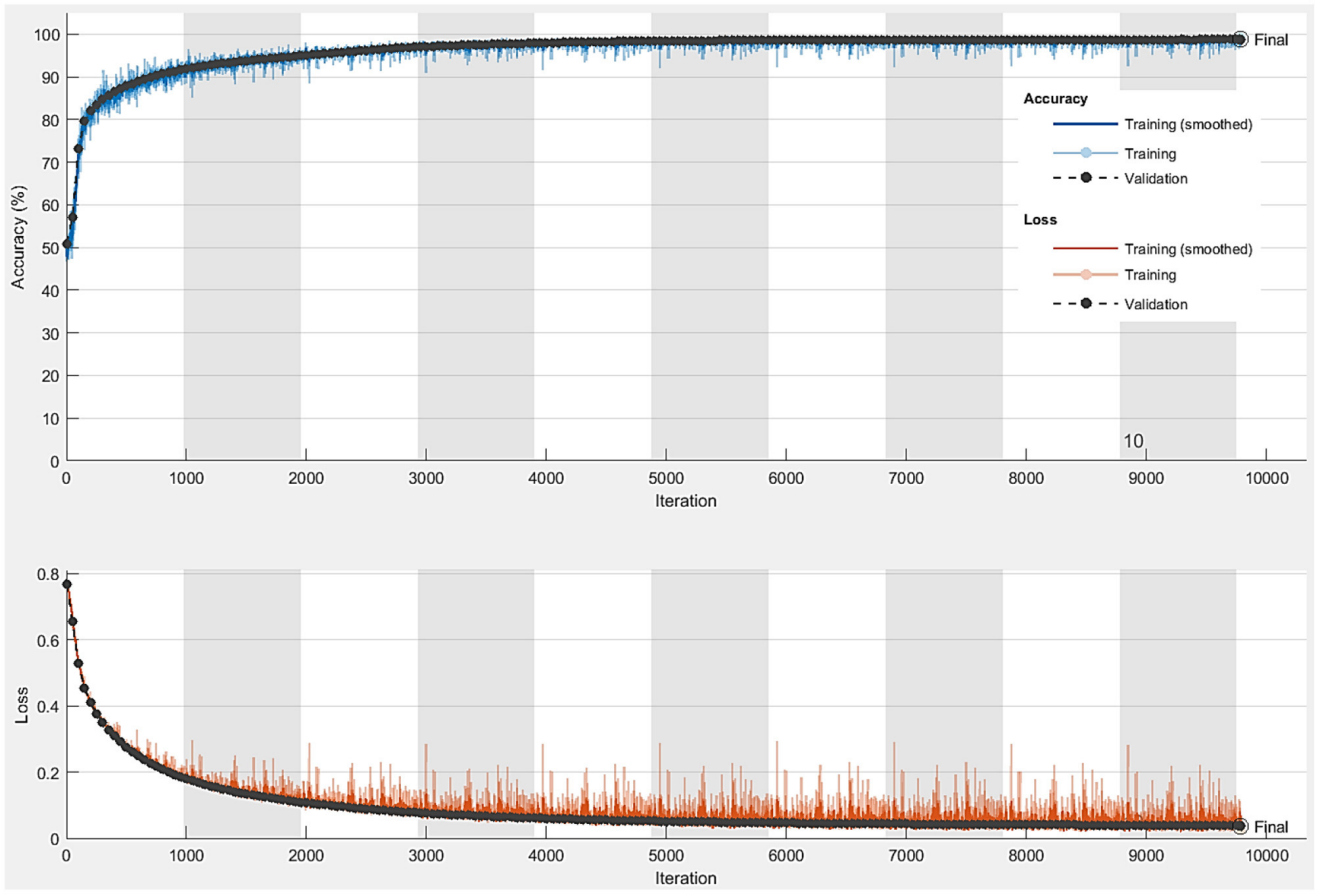
Accuracy and loss graph obtained at the end of training the SegNet network.

After the data augmentation step is completed, the total number of semantic data is 3,000. The number of data is more suitable for SegNet architecture. The size of each original and mask images is resized to 600 × 400 to reduce the training time. The SegNet architecture is able to train end-to-end thus ensure all the weight in the network to be more optimized and efficient. In addition, SegNet architecture consists of a hierarchy of decoders where one corresponded to each encoder. 95% of the total of 3,000 images obtained after data augmentation in the Semantic Drone Dataset is reserved for training. Our shopping mall dataset will be used for the real-time application, so the training rate for the public dataset is high. In this way, it is aimed to obtain a robust network trained with more image data. The hyperparameter values that must be adjusted for training within the SegNet architecture are shown in Table 2. In Table 2, it is seen that the Mini Batch value that enables the separation of training data into small groups is 32 and the optimization algorithm that reduces the training error by adjusting the weight values is Stochastic Gradient Descent with Momentum (SGDM). The accuracy and loss graph obtained after the training is completed is shown in Figure 10. According to the graphic, the pixels of the training and test images are classified with high accuracy. Classification accuracy for test images is 98.86%. Although high accuracy is obtained from the SegNet architecture, it was developed based on the Semantic Drone Dataset. Since this study focuses on the detection of human presence in the mall, it is important for the network to be trained to semantically distinguish people in the UAV images in our own dataset.

**Table 2:** For the training of the SegNet network, the parameters determined before the training.

**Optimization algorithm**	**Maximum epoch**	**Mini batch size**	**Learning rate (**α**)**	**Momentum (γ)**
**Training parameters**				
SGDM	25	32	0.001	0.95

**Figure 11:**
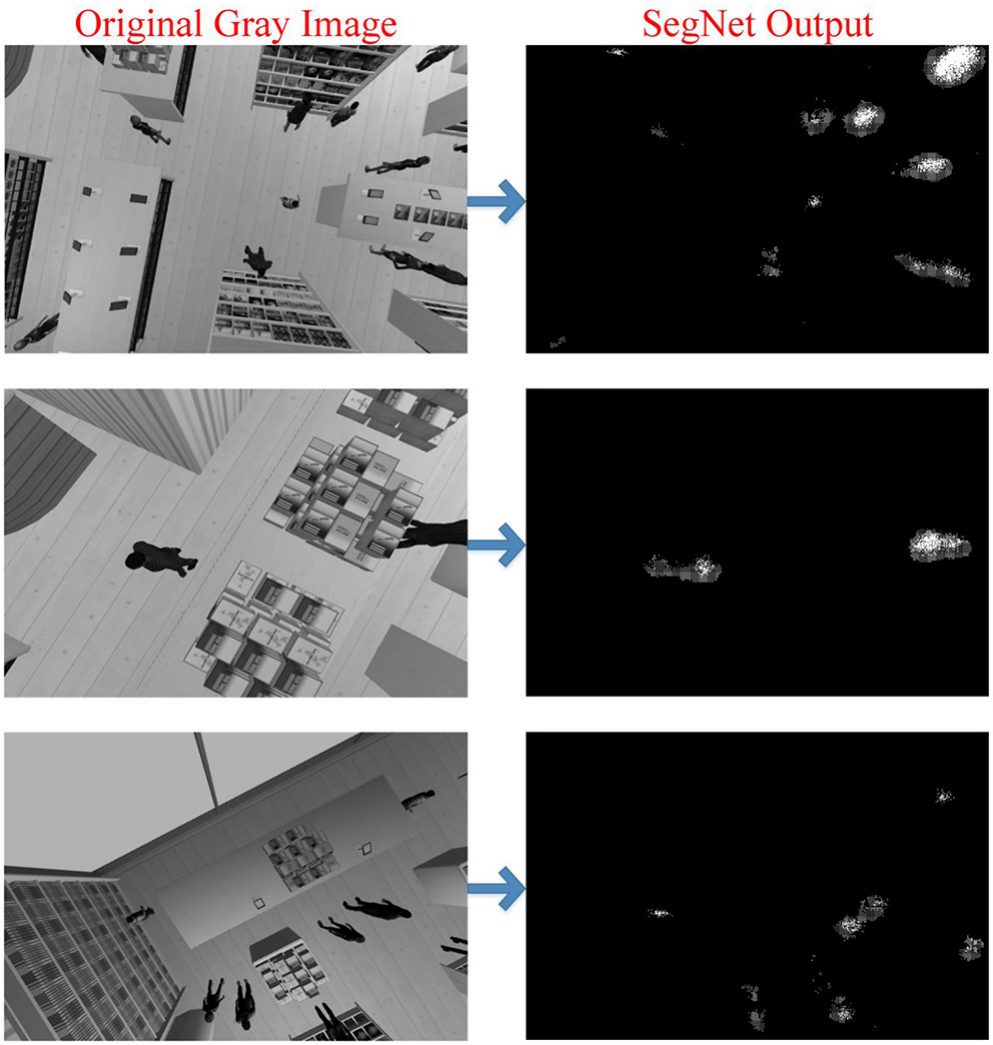
Some frames of the Shopping Malls Dataset (Original Gray Image) and semantic human masks obtained as a result of feeding these frames to the trained SegNet (SegNet Output).

**Figure 12:**
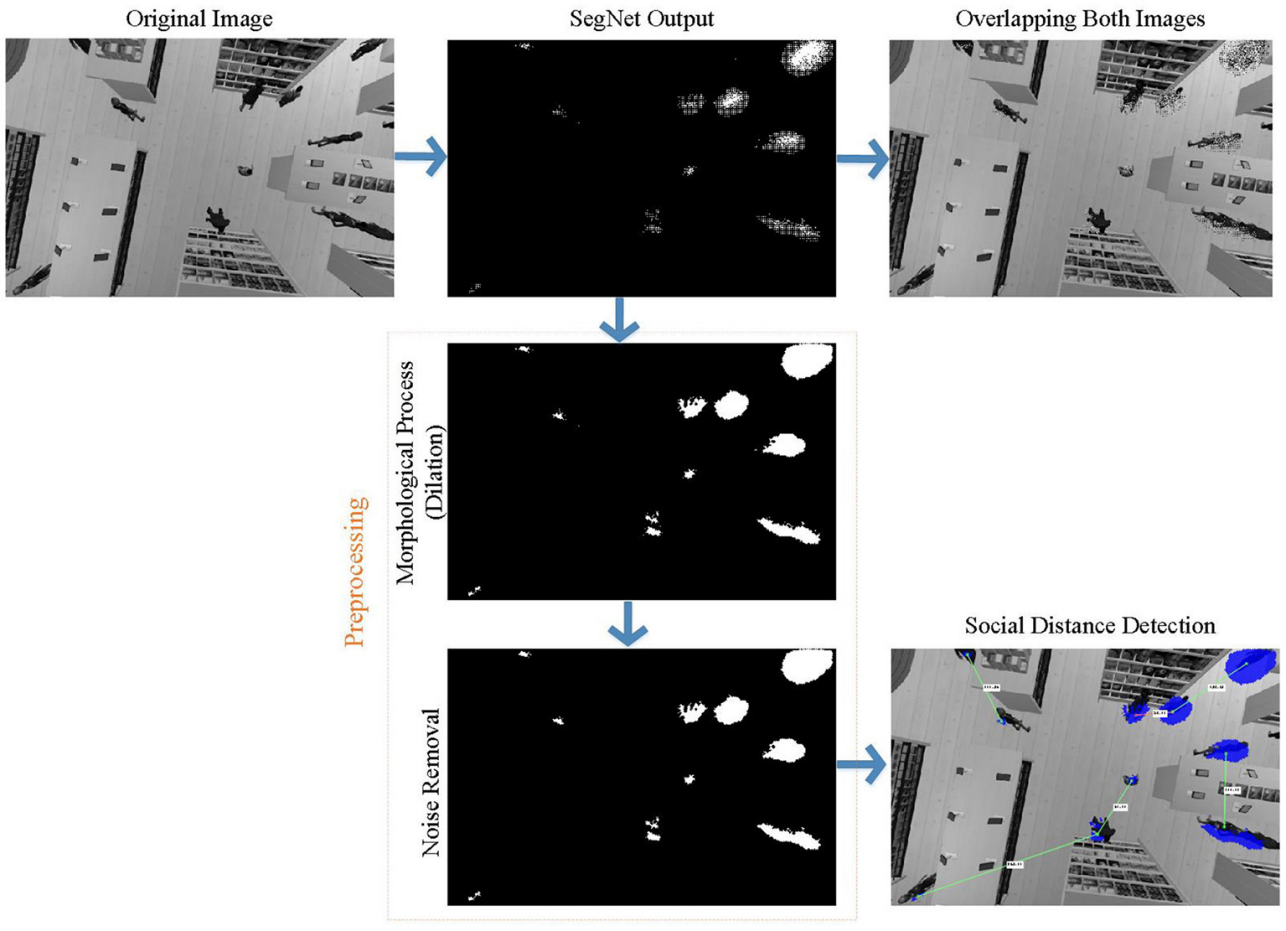
Implementation of image preprocessing steps and social distance measurement in a frame of the shopping malls dataset (Trained SegNet misclassifies some pixels for the shopping mall dataset. These pixels are removed with preprocessing so that these defective pixels are ignored for social distance measurement. The mask image where noise is removed is used for social distance measurement. The distance line is red where social distance is violated).

Once the training is completed, the mask images obtained from the trained SegNet network are given in Figure 11. Although the results are promising, some pixels are classified incorrectly due to pixel-based classification as shown in Figure 11. With the subsequent image preprocessing steps, incorrectly classified pixels are removed as shown in Figure 12. As can be seen in this figure, the discrete pixels formed surrounding human features should be combined. This is because the pixels collected in a certain local area represent a single person. Morphological process (dilation) is applied to solve this issue. In the next and last preprocessing step, single pixels (misclassified) formed in different regions in the SegNet output are removed. At the end of these two steps, preprocessing is completed. The figure also indicates the result of semantic human detection. These preprocesses are applied to all frames to ensure more accurate and robust human detection can be achieved, as shown in Figure 12. After a successful person detection, the distances between two people closest to each other are calculated. Normally, social distance lines are green, while the line is denoted as red when the social distance rule is neglected (Figure 12).

## Conclusion

Although the COVID-19 are nearing endemic, its impacts has disrupted our lives due to the speed of transmission, mutation of the virus, etc. Therefore, in order to overcome this pandemic, we need to follow rules such as cleaning, masking mandate and social distancing in our social life. Motivated by this issue, this study aims to reduce the transmission of infection between people in shopping malls where people are crowded during the COVID-19 period. Of course, the aim is to reduce the spread of the infection by producing a solution to the pandemic in crowded environments. In other words, the proposed method can also be applied to different places such as airports, libraries and large restaurants. The proposed application enables people to reduce contact with shopping carts and maintain distance between people through both a ground robot and a UAV. The ground robot acts as a shopping cart that follows the customer while shopping. Through this way, customers will have shopping experience in more comfortable and cleaner conditions. In addition, this provides great convenience for elderly people. With a ground robot with a monocular camera, the measurement of social distance between customers is often inaccurate due to the camera angle. A bird's-eye view is more reliable for measuring the distance between two people. For this purpose, it is ensured that customers who shop in the shopping center are detected by a UAV that travels independently of the ground robot. Places where the distance between people is < a certain threshold value can be determined in this way.

For the application of the proposed method, monocular images obtained from the front camera of the ground robot and the bottom camera of the UAV are used. First human detection and then human tracking should be performed with the ground robot. The human in the frame is detected with the HOG-SVM method, which enables successful identification and classification of human by making use of human shape features. In the next step, the detected human is followed up with Bayesian-based KF. In order for the UAV to perform its mission, it must detect people taken from the bottom camera. However, it would not make sense to use HOG for this, because the shape features of human beings vary greatly in the images taken from the bottom camera. Therefore, instead of HOG, semantic segmentation, which is a more modern detection method, is applied with SegNet architecture. However, this requires training with a dataset containing UAV frames and semantic masks. The Semantic Drone Dataset is utilized in this study. Since this study only requires two classes (human, background) and the original dataset contains 20 different classes, we performed data conversion and augmentation to increase the number of data. The SegNet achieved test accuracy of 98.86%. Then, this trained network is applied to our shopping malls dataset and several preprocessing steps are applied to the binary mask images in the SegNet output. Finally, the human positions are detected more accurately and social distance between people is measured. This study aims to maintain social distance between customers and reduce contact between people in a crowded environment. The promising results from this study shows that an automated system can be employed to reduce the risk of COVID-19 transmission between humans. The study proves that human intervention can be minimal and constant supervision can be minimized. Therefore, it is necessary to develop an automated system. This study differs from previous studies in terms of considering both contact and social distance together. Moreover, the results show that such a system is feasible.

## Discussion and Future Works

This study proposes multi-robots in crowded places such as shopping malls to reduce the spread of the COVID-19 crisis. It is the first study to address both contact and social distance with a multi-mobile robot. Therefore, the proposed method has its shortcomings and needs to be improved. First of all, this study was not carried out in a real-world environment, it was applied in a simulation environment produced in the ROS Gazebo. Real-world applications are more complex and require solving different challenges. For example, the ground robot may encounter different obstacles due to its design, in this case, the robot's behavior should be determined. The design of the robot is very important. In addition, occlusion is likely to occur during human detection, even humans may not be detected due to different illumination, noise, etc., and the tracking algorithm should be able to cope with these situations. The KF-based tracking used in this study can produce a prediction if the human is not detected, but this prediction needs to be developed. Finally, it is possible to detect more than one person in human detection with the ground robot, and this problem should be solved.

Although there are many shortcomings that need improvement, successful results in the simulation environment proved that this study can be developed and replicated in a real environment in the future. This is because, the dataset used is created in a similar manner mimicking a real shopping mall. The basic problems described above will guide our future work. In particular, the application will be implemented in a real shopping center. In this way, it is aimed to solve different problems encountered. As an innovation, a thermal camera can be embedded to the ground robot to detect if the person in the indoor environment has fever. It is also planned to determine whether people are wearing masks or not by using the ground robot. Finally, wireless charging stations for charging the batteries of ground robots are the subject of future studies.

The future work plan for UAV is autonomous capability. Recently there have been advances in studies on autonomous UAVs. To solve the autonomous UAV problem, Simultaneous Localization and Mapping (SLAM) should be addressed. Thanks to SLAM, it is planned that the UAV will map the shopping center and make autonomous path planning according to this shopping center map. Of course, in this case, the main problem for the UAV will be the battery. Finally, we will create our own semantic UAV dataset for a more successful semantic segmentation.

## Data Availability Statement

The raw data supporting the conclusions of this article will be made available by the authors, without undue reservation.

## Author Contributions

MAs, AY, AD, and KS designed and developed the study protocol as well as major contributors to the article writing. KH and MAz are involved in results and algorithm verification. All the authors checked all the synthesized data and approved the final version to be submitted for publication. All authors have substantially contributed to the article.

## Conflict of Interest

The authors declare that the research was conducted in the absence of any commercial or financial relationships that could be construed as a potential conflict of interest.

## Publisher's Note

All claims expressed in this article are solely those of the authors and do not necessarily represent those of their affiliated organizations, or those of the publisher, the editors and the reviewers. Any product that may be evaluated in this article, or claim that may be made by its manufacturer, is not guaranteed or endorsed by the publisher.

## Footnotes

^1^ https://www.youtube.com/watch?v=SJGH53AfwvA

^2^ https://www.ros.org
